# The Effect of Displacement Constraints on the Failure of MEMS Tuning Fork Gyroscopes under Shock Impact

**DOI:** 10.3390/mi10050343

**Published:** 2019-05-24

**Authors:** Jiangkai Lian, Jianhua Li, Lixin Xu

**Affiliations:** School of Mechatronical Engineering, Beijing Institute of Technology, Beijing 100081, China; 2120170227@bit.edu.cn (J.L.); lxxu@bit.edu.cn (L.X.)

**Keywords:** MEMS tuning fork gyroscope, displacement constraint, failure, shock resistance

## Abstract

Displacement constraints such as stops are widely used in engineering to improve the shock resistance of microelectromechanical system (MEMS) tuning fork gyroscopes. However, in practical applications, it has been found that unexpected breakage can occur on MEMS tuning fork gyroscopes with stops. In this paper, the effects of two displacement constraints on the failure mode of MEMS tuning fork gyroscopes are studied. The MEMS tuning fork gyroscope is simplified to a two-degree-of-freedom (2DOF) model, then finite element analysis (FEA) is used to study the effects of displacement constraint on the gyroscope. The analysis proves that even if the displacement constraint of direct contact with the weak connecting beam is not established, the equivalent stiffness of the gyroscope can be enhanced by limiting the displacement of the movable mass, thereby improving the shock resistance of the gyroscope. However, under the shock of high-g level, displacement constraint with insufficient spacing will cause multiple collisions of the small-stiffness oscillating frame and lead to an increase in stress. The cause of failure and shock resistance of a MEMS tuning fork gyroscope are verified by the shock test. By comparing the results, we can get a conclusion that is consistent with the theoretical analysis.

## 1. Introduction

Microelectromechanical systems (MEMS) tuning fork gyroscopes have the advantages of low cost and high integration, and thus are widely used in various categories such as consumer electronics, automobiles, precision munitions, and artilleries [1,2,3,4]. When used in precision munitions and artilleries, the survival of a MEMS tuning fork gyroscope under the shock of a gun launch is concerned. During the gun launch, the sensor is subjected to high mechanical shock with peak acceleration up to 10,000–20,000 g. A gun shock consists of a long shock (>5 ms) with some high-frequency shock components (<200 µs) [4,5]. Moreover, the duration of the shock pulse, made by high-g test equipment, ranges from tens of microseconds to hundreds of microseconds [6,7,8,9,10]. In order to avoid failures, whether shortening the distance between the movable silicon structure and the substrate, or adding a stop structure, displacement constraint is widely used as an effective method in engineering. However, under these high-g shock impacts of short pulse duration, even with displacement constraint, MEMS tuning fork gyroscopes are still very fragile. 

Previous studies on the failure of MEMS tuning fork gyroscopes under shock impact have been carried out. The duration of the shock pulse has been the concern of some articles. Srikar et al. [11] proposed a criterion for classifying the shock response in relation to the acoustic transit time and the duration of the shock load. They pointed out that most MEMS devices respond quasi-statically in shock environments. Research conducted by Younis et al. [12] proved that when the duration of the external shock is much larger than the natural period of the structure itself, the duration of the shock pulse has little effect. Sundaram et al. [13] calculated that the system is most prone to damage when the natural period approaches to the duration of shock pulse. They considered that failure can occur when the displacement of the mass reaches the maximum displacement required for failure. Zhang et al. [14] proved that shock impacts in different directions result in different positions of failure. The maximum displacement required for failure is believed to be a valid standard for design. Zhou et al. [15] pointed out that it is necessary to limit the maximum displacement of the oscillating frame. Jiang et al. [16] presented the idea that a stop can effectively improve the shock resistance of MEMS devices. The experiment made by Li et al. [17] proved that if the proof mass is severely displaced, it can result in damage. Further, the effects of external components (such as the Printed Circuit Board (PCB), package, etc,) on MEMS tuning fork gyroscope failure have been investigated. The exploration work of Ghisi et al. [18] indicated that the package would deform and store energy when impacted, which could make MEMS structures vulnerable. The research of Alsaleem et al. [19] shows that when the PCB experiences a dynamic load it will significantly magnify the effects of the shock impact as the frequency of the PCB approaches that of the microstructure. 

Some progress has been made both on the MEMS die and external components. In summary, when the impact pulse duration is close to the natural period of a system, a MEMS tuning fork gyroscope is most likely to fail, and external components may amplify the magnitude of the impact. Limiting displacement can prevent failure when a MEMS tuning fork gyroscope is impacted. However, the motion of a MEMS structure with displacement constraints can couple a variety of factors. In turn, its response and stress under impact may exceed expectations. The displacement constraint changes the characteristics of the motion that MEMS structure originally had and forms a new system that interacts with the MEMS structure when the structure is impacted. In the response of MEMS tuning fork gyroscopes when subjected to the shock impacts in different directions, it is necessary to further study the effect of displacement constraints on MEMS tuning fork gyroscopes. The cause of MEMS tuning fork gyroscope failure from displacement constraint under shock impacts needs to be clarified, and this could help to establish a theory to guide the design of MEMS tuning fork gyroscopes. 

In this paper, the effect of two displacement constraints on the failure of MEMS tuning fork gyroscopes under shock impacts in different directions is studied. In Section 2, MEMS tuning fork gyroscopes are simplified to a two-degree-of-freedom (2DOF) model. An equation about deformation of the movable parts under shock loads is given, which theoretically explains the effect of displacement constraint on the failure of MEMS tuning fork gyroscopes under shock impacts. In Section 3, finite element analysis (FEA) is applied to study the causes of failure of a MEMS tuning fork gyroscope under shock impacts with different pulse durations, as well as to study the effects of contact and non-contact displacement constraints on the gyroscope. And in Section 4, MEMS dies are impacted by a machete hammer in three orthogonal directions (X, Y, and Z). Test results are highly consistent with the theoretical analysis.

## 2. Theory and Equations

**Figure 1:**
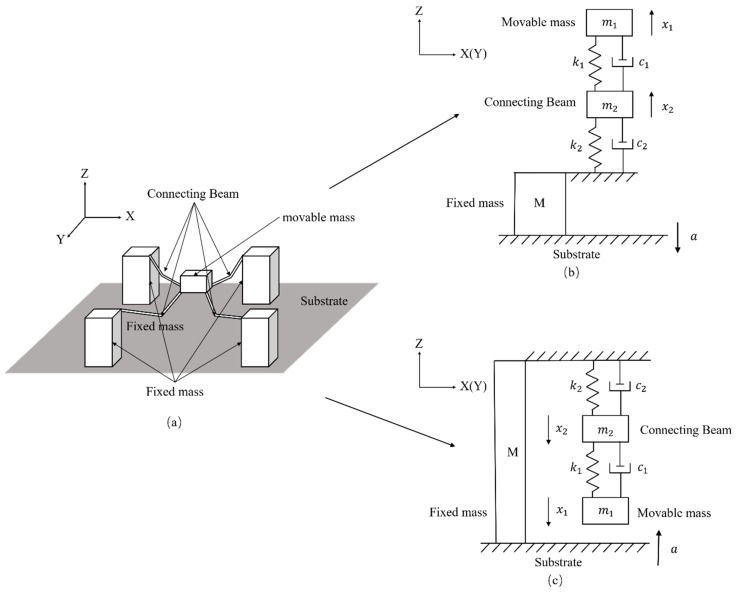
A two-degree-of-freedom (2DOF) model of a microelectromechanical systems (MEMS) tuning fork gyroscope. (**a**) Simplified schematic of the two-degree-of-freedom (2DOF) model; (**b**) schematic view of motion and stress of the model when the system is impacted along the negative Z-axis; (**c**) schematic view of motion and stress of the model when the system is impacted along the positive Z-axis.

A MEMS tuning fork gyroscope can be approximated as a 2DOF system. Figure 1a shows the schematic view of the 2DOF system, where *m* is the effective mass of the movable MEMS element, *c* is the effective damping coefficient, *k* is the effective stiffness, *x* is the displacement of the mass relative to the substrate when it was impacted, and *a* is the acceleration of the system. For convenience of description, the coordinate of the MEMS tuning fork gyroscope is added. Figure 1b,c show the schematic view of motion and stress of the model when the system is impacted along the Z-axis. 

The movable parts of a MEMS tuning fork gyroscope can be divided into two parts: the movable mass, and the connecting beam. For a MEMS tuning fork gyroscope with oscillating frames, its oscillating frames are often directly connected to the connecting beams (including coupling beams, decoupling beams, driving beams, and sensing beams). These connecting beams are connected to the movable and fixed masses. Therefore, the quality of the oscillating frame will be factored into the quality of the connecting beam. Even if the quality of the connecting beam is small relative to the movable mass, it cannot be ignored.

The movable parts of a MEMS tuning fork gyroscope will move upwards relative to the substrate and fixed mass when the system is subjected to a negative Z-axis shock impact. Following Srikar et al. [11], the response is described using a vibratory solution when the duration of the shock pulse is comparable to the time-period of vibrations. In this dynamic environment, the maximum displacement of the movable parts will exceed the maximum displacement of the movable parts in the quasi-static environment. Therefore, the displacement of movable parts without the stop under the shock impact is too large and the movable parts will be dangerous (see Figure 1b). More details of the displacement without the stop can be found in the [17]. Here, based on the 2DOF model, the motion of the movable mass is given by

(1) $$m_{1}{\ddot{x}}_{1}=m_{1}a+c_{1}{\dot{x}}_{2}-c_{1}{\dot{x}}_{1}+k_{1}x_{2}-k_{1}x_{1}$$

and the motion of the connecting beam is given by

(2) $$m_{2}{\ddot{x}}_{2}=m_{2}a-\left(c_{1}+c_{2}\right){\dot{x}}_{2}+c_{1}{\dot{x}}_{1}-\left(k_{1}+k_{2}\right)x_{2}+k_{1}x_{1}$$

**Figure 2:**
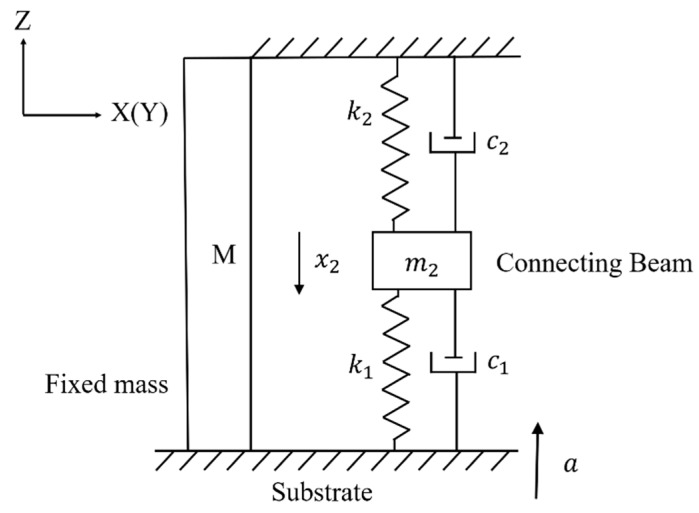
Model of a MEMS tuning fork gyroscope transformed from Figure 1c when the movable mass adheres to the substrate.

When a positive Z-axis shock impacts the MEMS tuning fork gyroscope, the movable parts will move toward the substrate. If the distance between the MEMS element and the substrate exceeds the maximum displacement of the movable parts under the shock impact, the motion and stress of the movable mass will be similar to the case where the gyroscope is subjected to the negative Z-axis shock impact. However, if the distance is less than the maximum displacement that the movable parts can take without stops, the motion and stress of the movable mass become different. In this situation, the movable mass and connecting beam will move toward the substrate, as shown in Figure 1c, and the movable mass will collide with the substrate during the duration of the shock pulse. If the velocity at the collision is not large, the movable mass will be almost stationary on the substrate. If the velocity at the collision is relatively large, the movable mass will rebound after the collision. The rebound can be seen as a reverse deceleration of the movable mass. However, when we focus on the motion of the connecting beam, it can be seen that the spring *k*_1_ is no longer stretched, regardless of whether the movable mass rebounds or rests on the surface of the substrate. As the shock impact is loaded, the connecting beam *m*_2_ continues to move downward, and the contribution of the spring *k*_1_ and the spring *k*_2_ to the restoring force increment of the connecting beam *m*_2_ is uniform. This is different from the contribution of spring *k*_2_ to the incremental force of the connecting beam *m*_2_ in Figure 1b,c. Therefore, when the movement of the movable mass is blocked and the force is provided by the substrate, the mechanical model of the connecting beam can be simplified, as shown in Figure 2. Figure 2 shows the situation in which the movable mass *m*_1_ is stationary on the surface of the substrate after the collision. If the movable mass rebounds, the length of spring *k*_1_ will become smaller here. The inertial acceleration required for the movable mass is converted to the support provided by the substrate. The motion of the connecting beam when the movable mass adheres to the substrate is given by

(3) $$m_{2}{\ddot{x}}_{2}=m_{2}a+\left(c_{1}+c_{2}\right){\dot{x}}_{2}+\left(k_{1}+k_{2}\right)x_{2}$$

Comparing Equations (2) and (3), the motion of the movable parts changes when the displacement of the movable mass reaches the limited maximum value. When the inertial force required for the movable mass is provided by the substrate, the risk factor in the system is on the connecting beam. The connecting beam will move further when it is subjected to an external shock impact, but the acceleration of the connecting beam ${\ddot{x}}_{2}$ will be reduced. This means that even if the substrate does not come into contact with the connecting beam, the shock resistance of the tuning fork gyroscope can be improved by blocking the movement of the movable mass that has the maximum deformation displacement. 

The natural frequency of the 2DOF undamped model $\omega_{n}$ is given by

(4) $$\omega_{n}=\sqrt{\frac{\frac{k_{1}+k_{2}}{m_{2}}+\frac{k_{1}}{m_{1}}}{2}\mp \sqrt{{\left(\frac{\frac{k_{1}+k_{2}}{m_{2}}-\frac{k_{1}}{m_{1}}}{2}\right)}^{2}+\frac{k_{1}^{2}}{m_{1}m_{2}}}}$$

and the natural frequency of the 1DOF undamped model when the movable mass adheres to the substrate $\omega_{n}^{\prime }$ (see in Figure 2) is given by

(5) $$\omega_{n}^{\prime }=\sqrt{\frac{k_{1}+k_{2}}{m_{2}}}$$

Take an example to study: If $k_{1}=k_{2}=k$, $m_{1}=2m$, $m_{2}=m$, then $\omega_{n}\approx \sqrt{\frac{k}{4m}}$ and $\omega_{n}^{\prime }=\sqrt{\frac{2k}{m}}$. If the ratio of $m_{1}$ to $m_{2}$ is further increased, then $\omega_{n}$ will be much larger. It can be seen that even if the connecting beam is not directly blocked, the natural period of the entire system changes, since the limited maximum displacement of the movable mass is constrained. The equivalent spring stiffness increases, and the equivalent mass decreases, resulting in an increase in the shock resistance of the system. If the displacement constraint is set in consideration of the maximum displacement of the connecting beam, the distance between the substrate and the suspended MEMS component will be excessively shortened, resulting in manufacturing technical difficulties and adhesion of the component to the substrate [20,21,22,23,24]. Increasing the natural frequency of the gyroscope is a well-known method of improving the shock resistance. However, increasing the operating frequency may mean increasing the stiffness of the connecting beam, which means that its sensitivity is reduced. By setting the displacement constraint to prevent the movement of the movable mass, the stiffness of the MEMS tuning fork gyroscope under the shock impact can be improved without changing the sensitivity of the gyroscope under normal working conditions, thereby improving the shock resistance of the gyroscope.

**Figure 3:**
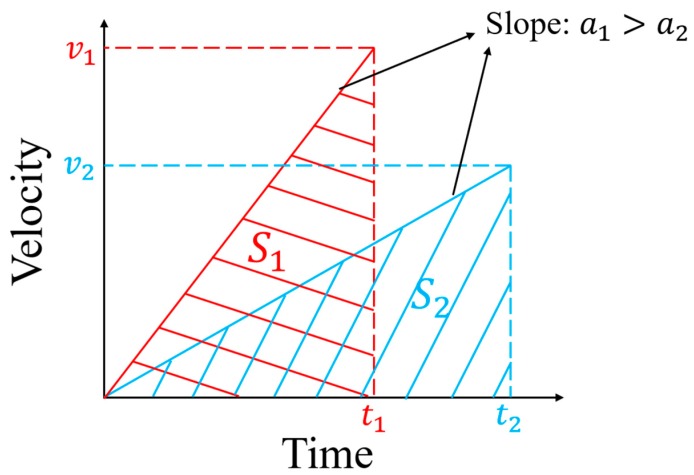
Comparison of two acceleration processes. A higher loading rate of shock impact means greater acceleration at each moment, which that means a higher velocity at the collision.

If the shock impact is parallel to the X/Y plane, the oscillating frames will easily collide with the stops on either side, whether the stops are added stops or the mass of the MEMS element. If the displacement of the mass without stops exceeds the limited distance when impacted, a collision will occur between the oscillating frames and stops, and the stress caused by collision may be enormous. The value of the stress is closely related to the velocity at the collision and the shape of the structure. In terms of the velocity at the collision, the higher the velocity, the higher the stress. Take an example of the process with a constant value of acceleration (see Figure 3). When the distance is constant ($S_{1}=S_{2}$), a large relative acceleration ($a_{1}>a_{2}$) will make the velocity at the collision higher ($v_{1}>v_{2}$). A shorter duration of the shock pulse represents a higher loading rate of the shock impact, which means that the oscillating frames will have greater acceleration relative to the substrate at each moment. As a result, it will cause a more serious collision.

## 3. Simulation and Analysis

### 3.1. FEA Model and Setting

**Figure 4:**
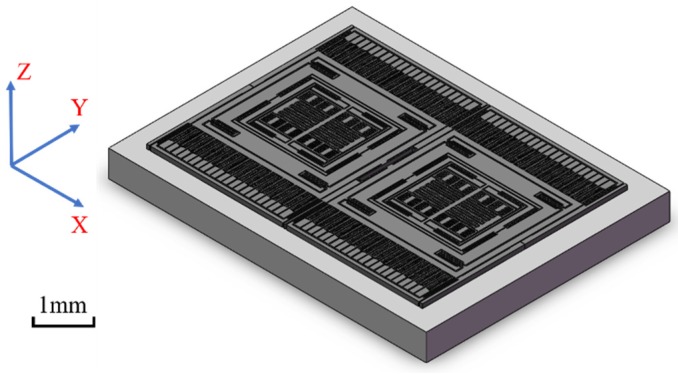
Schematic view of a MEMS tuning fork gyroscope.

The structure of a MEMS tuning fork gyroscope is shown in Figure 4. The single-axis MEMS tuning fork gyroscope is made by bulk micromachining, which is made of single crystal silicon. The MEMS tuning fork gyroscope includes movable parts, surrounding fixed mass, and combs. The surrounding fixed mass and combs are bonded to the borosilicate glass substrate. The connecting beam connects the movable parts and the fixed mass.

Finite element analysis (FEA) was used to analyze the performances of a MEMS tuning fork gyroscope structure under high mechanical shock. Here, the transient structural module in ANSYS Workbench 16.0 was applied to analyze the mechanical response of the gyroscope to shock impacts. The sensor in the gun launch impacted from the bottom. Similarly, the hammer with the initial velocity stopped suddenly, resulting in a bottom-up stress wave. In order to keep the generality, a translational joint constraint was applied to the bottom of the borosilicate glass substrate to ensure that it only moved in the direction of the shock. The shock load will be applied to the bottom of the substrate.

**Figure 5:**
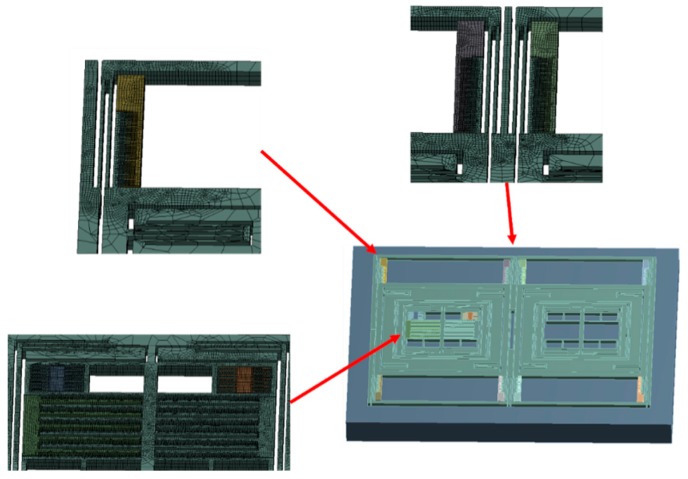
Simplified model and refined parts.

Considering that the comb and the slender beam were relatively small in size, finer mesh for both parts was needed. Further, to reduce the amount of calculation and accelerate the generation of results effectively, a simplified model was used. As shown in Figure 5, parts of the model were refined. In the model, the gyroscope components were meshed with 118,417 solid elements and 768,062 nodes. The large areas of vacancy in the model are comb structures that were repeated with the remaining comb structures. A small number of representative comb structures were retained to ensure the accuracy of the analysis. The retained combs ensured symmetry and typicality as much as possible. The contact settings of the parts that could collide were set to frictionless contact. 

The material properties [6] used are listed in Table 1. In the simulation, since single-crystal silicon is a brittle material, the ultimate strength is different in previous studies. More details on single-crystal silicon can be found in [25,26]. Taking the results of Wan et al. as a reference, the minimum stress of the sample fracture in their test was 0.67 GPa, and the fluctuation between the maximum value and the minimum value was large. The ultimate strength was related to the size of the sample and the test method. As quasi-static fracture strength is a valid criterion [11], first principal stress is used to assess the shock resistance of the MEMS tuning fork gyroscope. The first strength theory works well for brittle materials in explaining the phenomenon and predicting dangerous locations.

### 3.2. FEA Results and Discussion

In combination with FEA, a model of the MEMS tuning fork gyroscope was built to simulate the shock impact process. With the first strength theory, we assessed and even predicted structure failure by comparing the first principal stress and ultimate strength of a MEMS tuning fork gyroscope. Structural failure here refers to structural fractures, excluding stiction and particle blockage [6,13].

Based on FEA, different failure conditions and causes were discovered. The following content is divided into two parts for detailed analysis.

#### 3.2.1. Failure Analysis of MEMS Tuning Fork Gyroscope under Z-Axis Shock Impact

**Figure 6:**
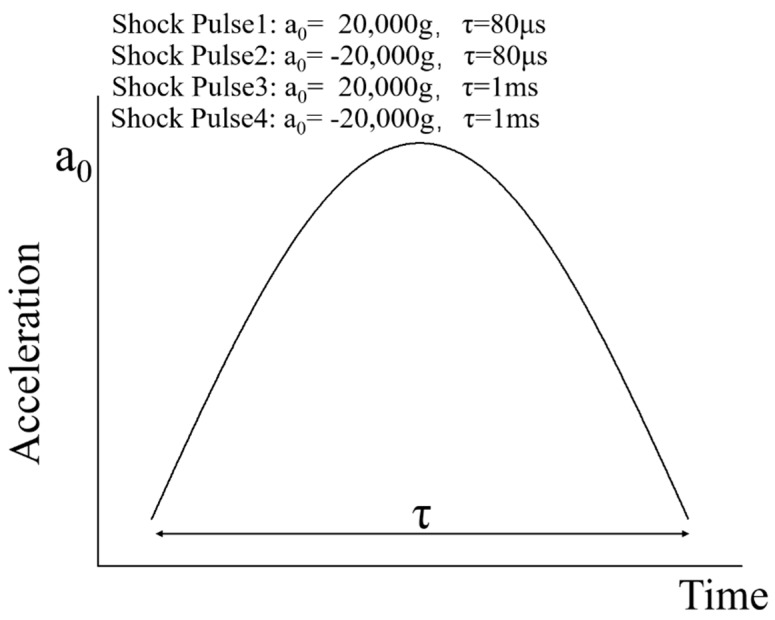
The Z-axis shock pulses of four different durations and directions applied in the simulation.

**Figure 7:**
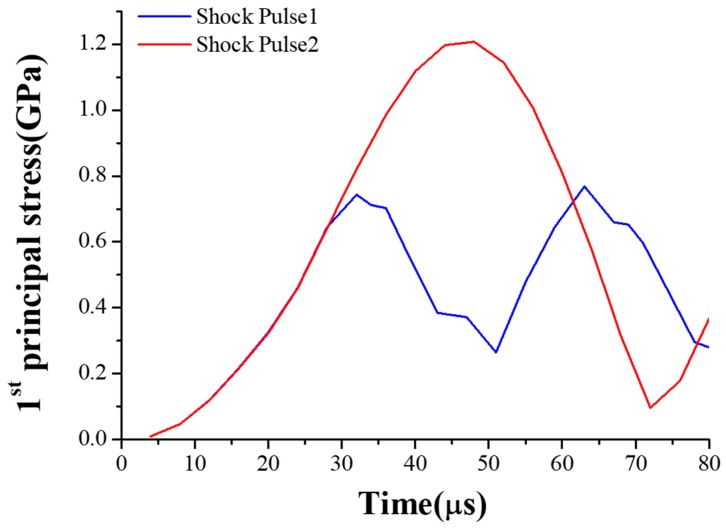
First principal stress of the MEMS tuning fork gyroscope under two Z-axis shock pulses with shock pulse duration of 80 μs.

**Figure 8:**
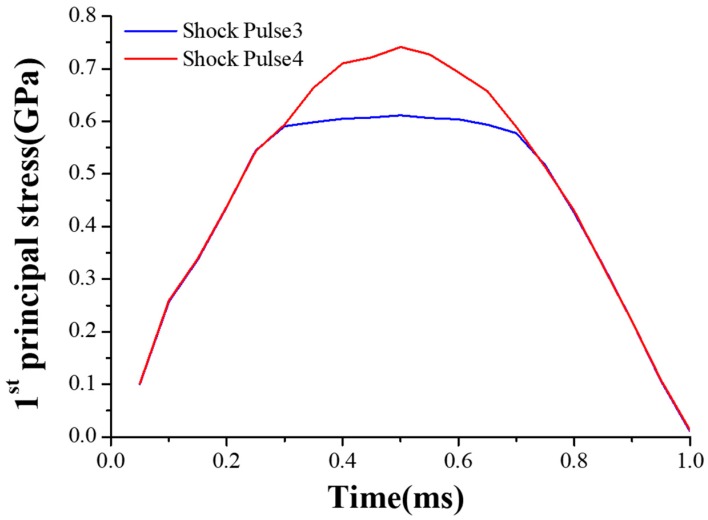
First principal stress of the MEMS tuning fork gyroscope under two Z-axis shock pulses with a shock pulse duration of 1 ms.

The MEMS tuning fork gyroscope was impacted by the shock pulses of four different durations and directions, using waveforms as shown in the typical pulse in Figure 6. Shock Pulse 1 and 2 simulated the shock impacts from the bottom to the top. Shock Pulse 1 was a positive Z-axis shock impact with a peak acceleration of 20,000 g and duration of about 80 μs. In contrast to Shock Pulse 1, the direction of Shock Pulse 2 was negative along the Z-axis. Its peak acceleration and pulse duration were the same as Shock Pulse 1. Compared with Shock Pulses 1 and 2, Shock Pulses 3 and 4 simulated the shock impacts at which the duration of the shock pulse is extended. Figure 7 and Figure 8 show the first principal stress of the MEMS tuning fork gyroscope under four Z-axis shock pulses.

**Figure 9:**
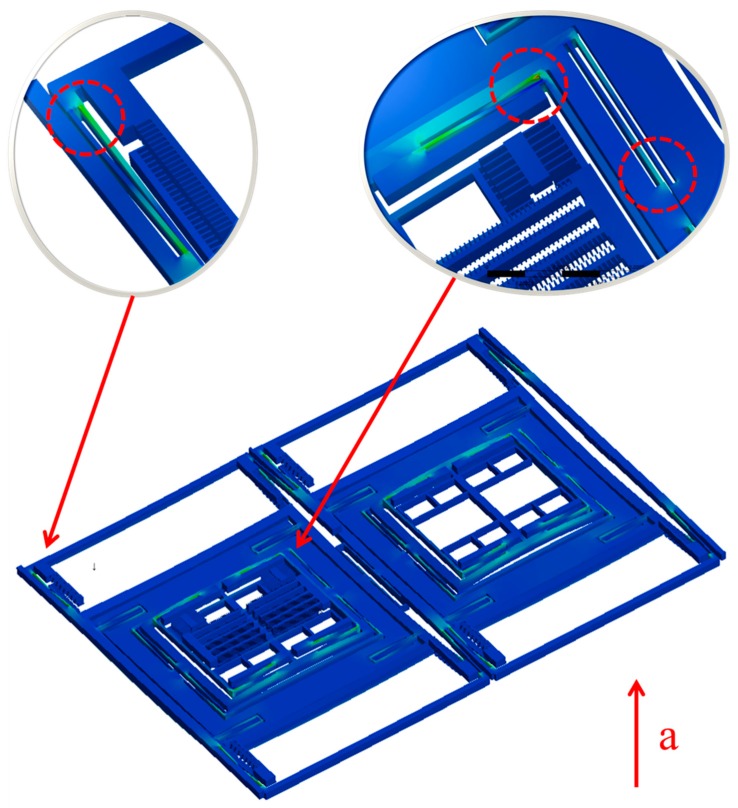
Deformation and weak positions under the impact of Shock Pulse 1 (the red arrows with ‘a’ indicate the direction of shock impact relative to the MEMS tuning fork gyroscope).

When the MEMS tuning fork gyroscope was subjected to both Shock Pulses 1 (Shock Pulse 3) and Shock Pulse 2 (Shock Pulse 4), the comparison of stress showed that the shock direction had a significant effect on the failure strength. In order to visualize the deformation of the internal structures, Figure 9 shows the impacted MEMS tuning fork gyroscope with an exaggerated deformation. The three weak positions in Figure 9 are due to the excessive bending deformation of the connecting beam, which led the root of the beam to be destroyed. The movable mass had the largest displacement. The connecting beam was deformed to a position between the fixed mass and the movable mass. It can be seen from the figure that the movable mass directly contacted or collided with the substrate, and the connecting beam did not come into contact with the substrate. As a result, the damage of the MEMS tuning fork gyroscope under the Z-axis shock impact was mainly due to excessive deformation of the connecting beam.

Comparing the stress of the MEMS tuning fork gyroscope under the impact of Shock Pulse 1 (Shock Pulse 2) and Shock Pulse 3 (Shock Pulse 4), it can be seen that even if the displacement constraint did not directly interact with the connecting beam, the shock resistance of the gyroscope was still improved. When the MEMS tuning fork gyroscope was subjected to the positive Z-axis shock impact, the maximum stress of the MEMS tuning fork gyroscope under the impact of Shock Pulse 1 was close to 0.8 GPa, and the maximum stress of the MEMS tuning fork gyroscope under the impact of the Shock Pulse 3 was close to 0.6 GPa. According to the modal analysis, the natural period of the silicon structure without considering any displacement constraint was about 60 μs along the Z-axis. As the duration of the shock pulse decreased close to the natural period of system, the peak stress with the displacement constraint was amplified by 33%. This enlargement resulted from the fact that the connecting beam had greater deformation when subjected to a stronger shock impact. 

However, when the MEMS tuning fork gyroscope was subjected to the negative Z-axis shock impact, the maximum stress of the MEMS tuning fork gyroscope under the impact of Shock Pulse 2 exceeded 1.2 GPa, and the maximum stress of the MEMS tuning fork gyroscope under the impact of the Shock Pulse 4 was less than 0.8 GPa. When other conditions were constant, the maximum stress of the MEMS tuning fork gyroscope without displacement constraint was amplified by more than 50% when the duration of the shock pulse was reduced (compare the maximum stress peaks when subjected to Shock Pulses 2 and 4, from 0.8 GPa to 1.2 GPa). These maximum stresses were all present at the root of the connecting beam, which were results of the large bending deformation. By comparing the magnifications of the stress peaks when subjected to Shock Pulses 1 and 3, and comparing the magnifications of the stress peaks when subjected to Shock Pulses 2 and 4, it can be seen that this indirect displacement constraint did change the natural period of the system under shock impact, even if the displacement of the connecting beam was not directly limited. This caused the displacement response of the weak part of the system (the connecting beam) to change, and indirectly improved the shock resistance of the MEMS tuning fork gyroscope. Although the weak position appeared on the connecting beam, the stiffness and natural frequency of the system under the shock impact were changed by limiting the displacement of the movable mass. For the connecting beam, this indirect displacement limitation avoided space restrictions and problems such as adhesion failure while facilitating the bonding process. The shock resistance of the gyroscope was effectively improved without changing the sensitivity of the working state. Similarly, such displacement constraints could be achieved by designing a local boss that sits beneath the movable mass. The non-contact displacement constraint avoids the direct collision between the connecting beam and the stop, and also avoids other troubles caused by the required small distance between the stop and the connecting beam.

#### 3.2.2. Failure Analysis of MEMS Tuning Fork Gyroscope under X-Axis/Y-Axis Shock Impact

**Figure 10:**
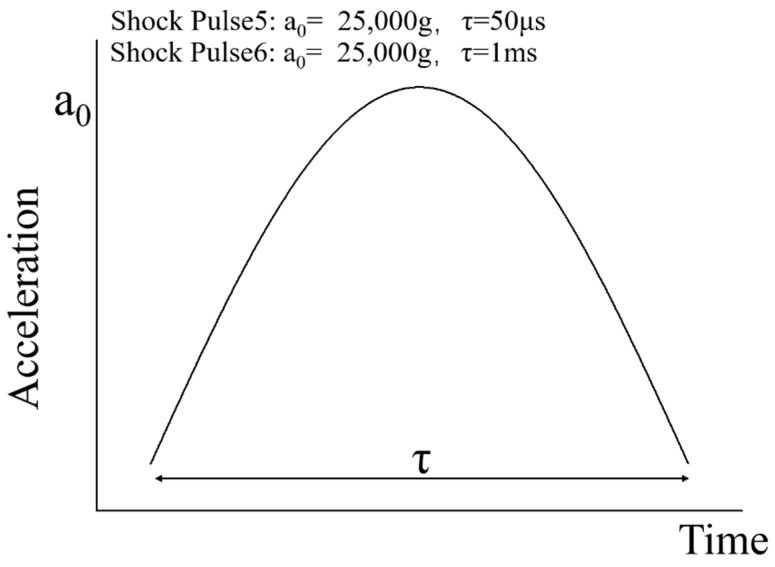
The X-axis shock pulses of Shock Pulses 5 and 6 applied in the simulation.

Similarly, as shown in Figure 10, the MEMS tuning fork gyroscope was impacted by three shock pulses along the X-axis direction. Shock Pulse 5 simulated a shock representative of high-g test equipment. The peak acceleration of Shock Pulse 5 was 25,000 g, and the duration of the shock pulse was 50 μs. In contrast, Shock Pulse 6 simulated a shock impact in which the duration of the shock pulse extended to 1 ms.

**Figure 11:**
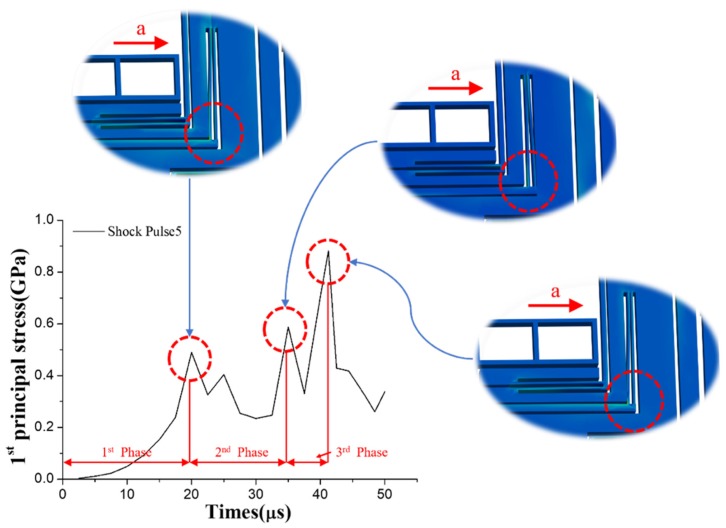
First principal stress of the MEMS tuning fork gyroscope and the deformation of weak position under the impact of Shock Pulse 5 (a_0_ = 25,000 g, τ = 50 μs, the red arrows with ‘a’ indicate the shock direction relative to the MEMS tuning fork gyroscope).

The dangerous position of the MEMS tuning fork gyroscope, when subjected to Shock Pulse 5, is shown in Figure 11. The result of the simulation showed that the maximum principal stress of the MEMS tuning fork gyroscope about 0.9 GPa. For the oscillating frame, the distance between the masses on both sides becomes its displacement constraint. The length of the oscillating frame is always long, and is nested between the masses. Therefore, when the MEMS tuning fork gyroscope is subjected to the X-axis shock impact, the oscillating frame tend to collide with the corners of the mass or combs. Since the beam of the oscillating frame is easy to move in the X-axis direction, when the shock with a short pulse duration is loaded, the end of the beam collides with the sharp edge of the mass. If the edge of this mass collides perpendicularly with the beam, the collision easily causes the surface of the contact point to break and the crack expands. And if the collision of the edge with the beam is a collision with a small angle from the side, the force generated by the collision will cause a shear stress at the root of the beam. At the root of A beam whose cross-sectional area suddenly decreases, the stress will be excessively stressed at the cross-sectional area to cause cracking. The crack will start from the root and tear the beam off the original structure.

There are three distinct peaks in the curve of Figure 11. The maximum principal stress of the MEMS tuning fork gyroscope was 0.9 GPa, corresponding to the third peak. The first peak was about 0.5 GPa, and the second peak was about 0.6 GPa. For the frame, the shorter the duration of the shock pulse, the higher the velocity of the collision between the frame and the stop on one side. Acceleration from relative rest to collision is considered the first phase. In the first phase, the external acceleration increases the relative velocity. From the knowledge of one-dimensional elastic collisions, when a small-sized oscillating frame hits a huge stop, the oscillating frame has a reverse velocity relative to the stop. Since the oscillating frame has a small mass and its structure is the slender beam, it is sensitive to the shock impact perpendicular to its length. In this direction (X-axis), the damping of the slender beam is small. At the same time, the distance limited by the displacement constraint on both sides of the frame is too small, meaning that the frame will collide with the stop on the other side. This process is considered the second phase, and external acceleration will reduce the relative velocity. Moreover, when the frame collides again and the velocity reverses, the third phase occurs, similar to the first phase. The difference between the first phase and the third phase of the frame is that the frame accelerates from an initial velocity in the third phase, and the external acceleration in this phase becomes large. Therefore, the relative velocity between the frame and the stop in the third phase will become higher than in the first phase. Furthermore, when a third collision occurs at the end of the third phase, the stress reaches the second peak, more intense than the first. 

The oscillating frame can be seen as a spring-mass system. The process of moving the oscillating frame from one side to the other can be seen as a process of moving the mass from the position where the spring is stretched to the position where the spring is compressed. And these two positions are symmetrical in regard to the balance position of the spring. During this movement, the mass is subjected to a restoring force of the spring and the shock load, which is in the direction of the compression of the spring. Generally, when the gyroscope is subjected to an impact, its oscillating frame will accelerate from the equilibrium position to one side. However, in the case of such a back-and-forth collision, this acceleration process needs to cover about twice the displacement of the one-way acceleration process. In addition, during the acceleration process, the acceleration of external shock impact may continue to increase. This results in a velocity at the collision that is much greater than the velocity at the collision when the collision occurs only on one side. Then the frame accelerates from one stop to the other. This causes the velocity at the collision when the frame accelerates from one stop to the other to be much greater than the velocity at the collision when the frame only accelerates from the equilibrium position to one side.

**Figure 12:**
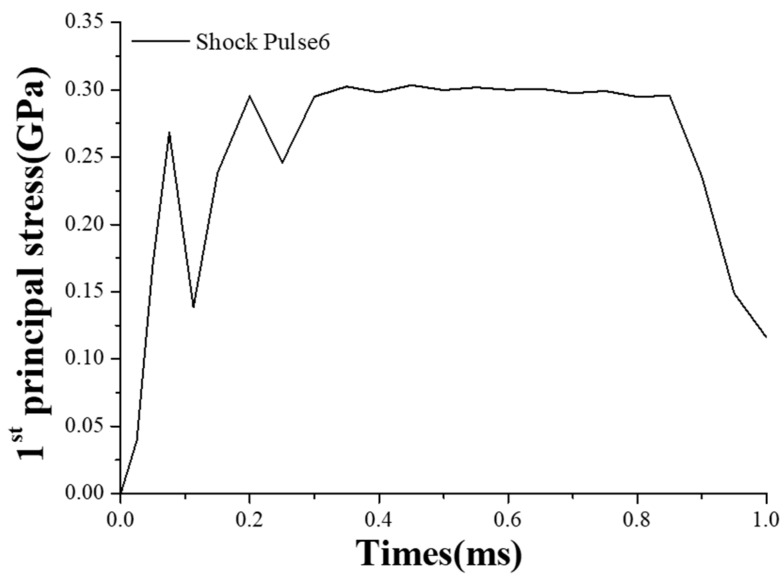
First principal stress of the MEMS tuning fork gyroscope under the impact of Shock Pulse 6 (a_0_ = 25,000 g, τ = 1 ms).

The results were different when Shock Pulse 6 impacted the MEMS tuning fork gyroscope. The duration of Shock Pulse 6 was extended, and the first principal stress was also reduced, as shown in Figure 12. From the curve, when the duration of the shock pulse was extended, the collision between the frame and the stop was also moderated. The frame did not repeatedly collide between the stops on both sides, as in the case of the frame impacted by the Shock Pulse 5. Under the shock impact of Shock Pulse 6, the frame chattered [27] after the collision with the stop. The amplitude of the chatter was gradually attenuated, and, finally, the MEMS tuning fork gyroscope was similar to a quasi-static force.

While Shock Pulse 5 and Shock Pulse 6 acted on the Y-axis, the stress of the MEMS tuning fork gyroscope was low enough to keep it intact. Since the sensitive direction of the oscillating frame did not follow the Y-axis, the substructure of the MEMS tuning fork gyroscope did not collide under the Y-axis shock impact. In the simulation, the MEMS tuning fork gyroscope did not fail when subjected to these two shock impacts.

According to the above analysis, when a MEMS tuning fork gyroscope is subjected to an X-axis shock impact, the primary cause of failure of the MEMS tuning fork gyroscope is the fracture of the oscillating frame caused by the collision. The distance between the stops in direct contact with the frame is small and the contact stiffness is large. Due to the sensitivity of the oscillating frame in this direction, a shock impact with a short pulse duration causes the frame to collide multiple times between displacement constraints. The collision is nearly elastic, which causes a rebound with the initial velocity after the collision. The process of moving from one side to the other side will couple the external shock impact. Thus, the acceleration process of the back-and-forth collision will cause the velocity at the collision to become larger than the acceleration process in which a collision occurs only on one side. In terms of the design of the spacing of the displacement constraints, the stress generated by the collision in this case needs to be considered. Otherwise, the inappropriate distance between the displacement constraints may make the gyroscope more vulnerable than the predicted result when subjected to a high-g shock impact.

## 4. Shock Test

### 4.1. Experimental Method

**Figure 13:**
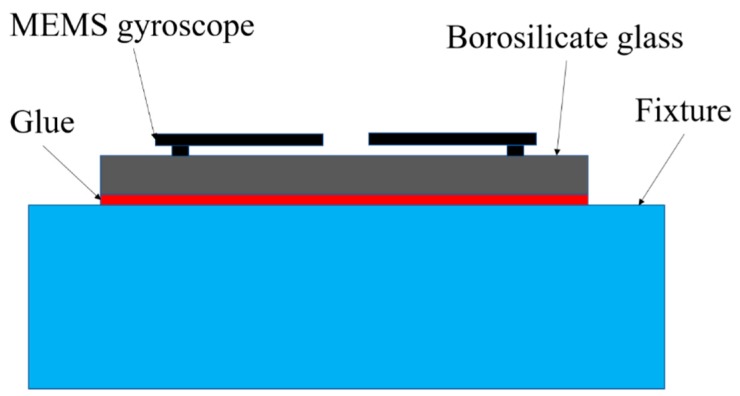
Schematic cross-section of the MEMS tuning fork gyroscope and fixture.

In order to avoid the effect of the package and bending deformation caused by solder bumps, the MEMS tuning fork gyroscopes were attached to the face of a cube fixture via a thin layer of glue (see Figure 13). This method ensured the rigid connection between the MEMS tuning fork gyroscope and the test fixture, avoiding the rigid collision of the components that can cause the secondary acceleration to be larger than the first.

**Figure 14:**
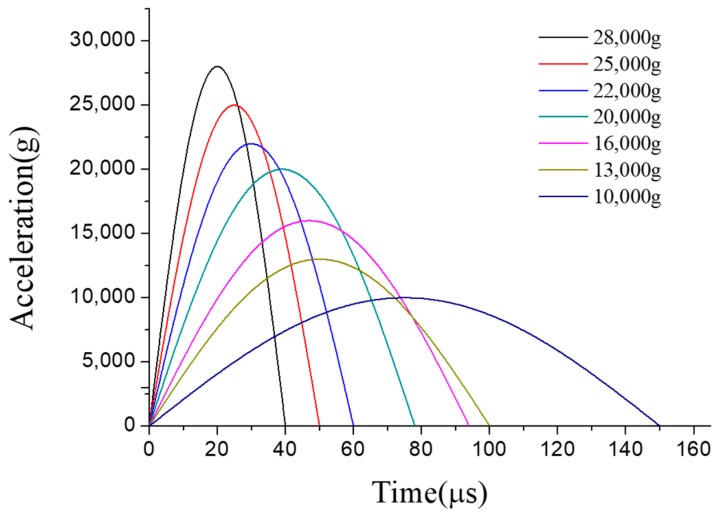
The fitted curves based on the measured acceleration pulses with different shock levels.

**Figure 15:**
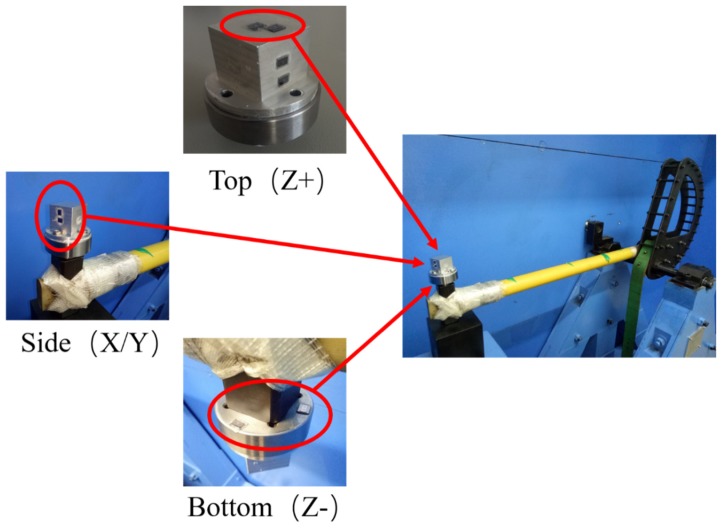
The experimental device and installation.

Machete hammers were used to produce a high-g shock. The device can produce an impact that simulates the mechanical shock of the gun launch. More details on machete hammers can be found in [9]. The fitted curves based on the measured acceleration pulses with different shock levels are shown in Figure 14. The cube fixture was glued to the hammer fixture via a thin layer of cyanoacrylate glue, which was screwed into the machete hammer again. The experimental device and installation are shown in Figure 15. The direction in which different MEMS dies were impacted is also identified in the Figure. The entire test process was done with the MEMS tuning fork gyroscope powered off.

In order to avoid cumulative damage [19,20], pre-tests were operated to determine the range of critical acceleration for the failure of MEMS tuning fork gyroscope. Then, new MEMS dies were used for the impact. The MEMS dies were glued to the surfaces of the cube fixture. Then, when the device was operated, the MEMS dies were impacted along the X/Y/Z axes simultaneously. The location of failure on the damaged sample was observed with a microscope after impact.

In order to be able to view the structure of the gyroscope directly with a microscope after the impact, and to avoid secondary damage to the structure of the gyroscope when the package is disassembled, the experiment was carried out in the air. This will inevitably add the effect of air damping on the experiment. The studies [13,17,18] show that air damping will reduce critical acceleration, but the law of the acceleration curve is consistent with the curve of the test result in vacuum. Therefore, critical acceleration corresponding to failure in the air may be slightly lower than he critical acceleration in a vacuum. However, by comparing all samples tested in the air, the effect of displacement constraint on critical acceleration can still be obtained.

### 4.2. Experimental Results

For comparison, MEMS dies were glued to various sides of the test cube. In order to make the results universal, the experiment used a total of 32 dies produced from the same batch for testing. These dies were equally divided into four groups, each of which was impacted in one direction. By changing the position and direction of the MEMS dies, they were subject to the shock impact in different directions. The experimental results are divided into the following two aspects.

#### 4.2.1. MEMS Tuning Fork Gyroscope Failure under Z-Axis Shock Impact

**Figure 16:**
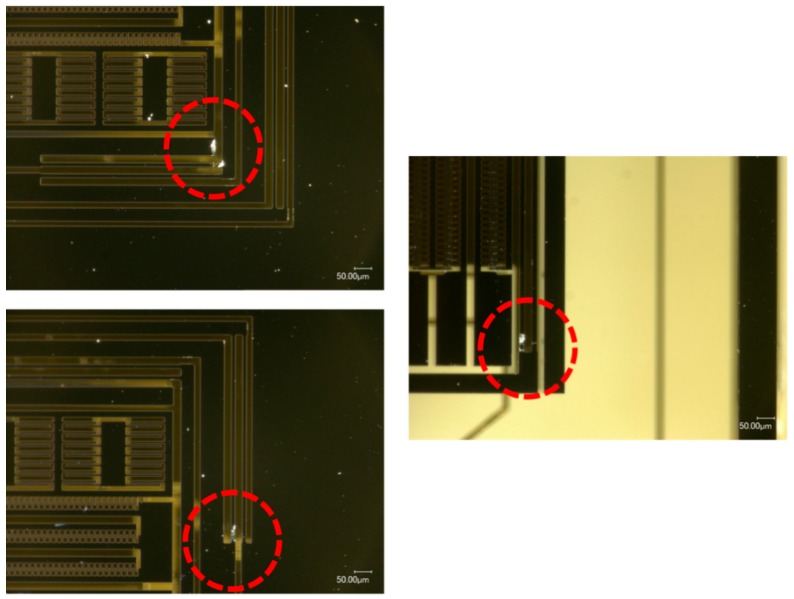
MEMS tuning fork gyroscope failures under positive Z-axis shock impact (a_0_ = 20,000 g, τ = 80 μs).

The borosilicate glass substrate was attached to the top and bottom surfaces of the test fixture to be impacted along Z-axis (see Figure 15). The MEMS dies were subjected to upward and downward shock impacts, respectively. When the silicon structure was subjected to a positive Z-axis shock impact from the substrate, damage occurred at the roots of the connecting beam. As shown in Figure 16, the MEMS tuning fork gyroscope failed under the positive Z-axis shock impact, in which the peak acceleration was about 20,000 g, and the duration of the shock pulse was about 80 µs (a_0_ = 20,000 g, τ = 80 μs). The oscillating frame and the connecting beam were broken under the shock impact.

**Figure 17:**
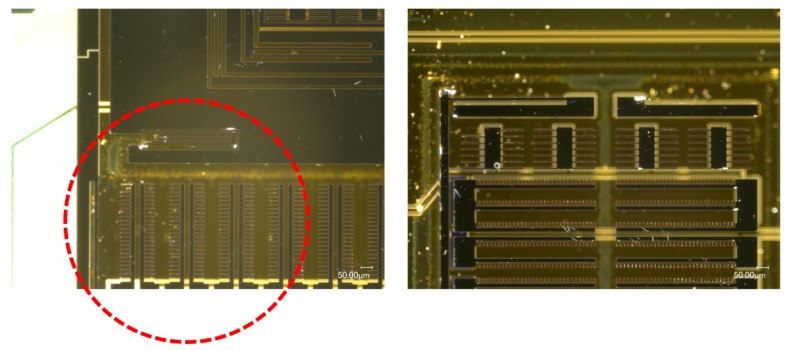
MEMS tuning fork gyroscope failures under negative Z-axis shock impact (a_0_ = 20,000 g, τ = 80 μs).

The MEMS dies stuck to the bottom of the test fixture were subjected to a negative Z-axis shock impact. Under the shock impact, in which the peak acceleration was about 20,000 g and the duration of the shock pulse was about 80 μs (a_0_ = 20,000 g, τ = 80 μs), the MEMS dies attached to the top surface were not damaged, but those attached to the bottom surface were destroyed entirely. The failure of these MEMS dies attached to the bottom is shown in Figure 17. It can be seen that the silicon structure peeled off in large areas. The beams and comb structures were generally broken.

When the MEMS tuning fork gyroscope was subjected to a negative shock, the critical acceleration of the failure was much smaller. When the MEMS tuning fork gyroscope was subjected to a negative Z-axis shock with a peak acceleration of about 15,000 g and a duration of about 100 μs, the connecting beam structure of the MEMS tuning fork gyroscope broke. The location of the fracture coincided with the location of the fracture in Figure 16.

Whether the MEMS tuning fork gyroscope was subjected to a positive Z-axis shock impact or a negative Z-axis shock impact, the fracture of the oscillating frame and the connecting beam was caused by the excessive bending of the connecting beam. Comparing the critical acceleration corresponding to the fracture, it can be seen that the distance between the substrate and the MEMS movable component exceeded the safe distance of the connecting beam, and the connecting beam broke as the acceleration of the external shock impact increased. However, the support of the substrate to the movable mass reduced the inertial forces acting on the connecting beam, increasing the stiffness of the system. Even if the substrate did not directly limit the displacement of the connecting beam, the indirect displacement constraint effectively improved the shock resistance of the gyroscope.

#### 4.2.2. MEMS Tuning Fork Gyroscope Failure under X-Axis/Y-Axis Shock Impact

**Figure 18:**
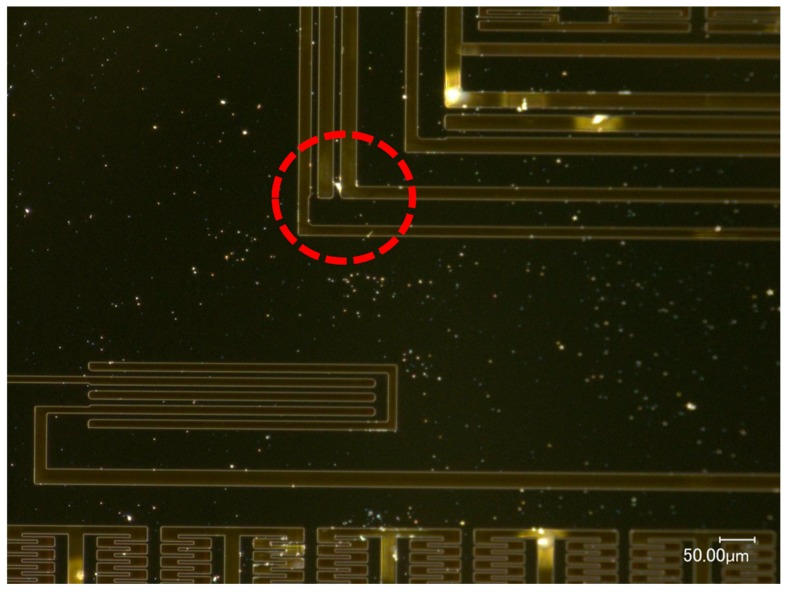
MEMS tuning fork gyroscope failures under X-axis shock impact (a_0_ = 25,000 g, τ = 50 μs).

When the MEMS tuning fork gyroscope was impacted along the X-axis, the damage occurred at the corner of the oscillating frame. As shown in Figure 18, under the X-axis shock (a_0_ = 25,000 g, τ = 50 μs), the oscillating frame was broken at the corner of the frame, and no significant breakage occurred elsewhere. Observing the incision orientation of the crack, it can be found that the crack caused by the shock impact started noticeably on the right side of the root of the beam and then spread along the edge of the beam contacting the mass until the beam was torn off. The slit of this crack was very consistent with the corner of the right mass, and the location of the fracture and critical acceleration were consistent with the simulation results. It can be shown that between such small-pitch displacement constraints, a small-stiffness oscillating frame will fail very easily due to the intense stress generated by the back-and-forth collision.

The MEMS tuning fork gyroscope exhibited high-g shock resistance when subjected to the Y-axis shock impact. Under the single maximum shock impact that the experimental device could produce, about 30,000 g, the MEMS tuning fork gyroscope still did not break.

It can be seen from the experimental results of the MEMS tuning fork gyroscope impacted by X-axis /Y-axis shocks that the MEMS tuning fork gyroscope exhibits high resistance to shock impact. When the MEMS tuning fork gyroscope is subjected to shock impact, the comb structures are relatively stable and do not collide. Instead, the dangerous locations are the roots of the slender beam and the oscillating frame, especially where the ends collide with the surrounding mass. It can be proven that the main cause of failure of the MEMS tuning fork gyroscope under X-axis shock impact was excessive stress at the end of the oscillating frame caused by collision. This excessive stress can lead to the generation and expansion of cracks in the root.

## 5. Conclusions

The displacement constraint has a significant effect on the failure of MEMS tuning fork gyroscopes in terms of cause of failure and change of shock resistance. These effects are also different when a MEMS tuning fork gyroscope is subjected to shock impacts with different pulse durations in different directions. When a MEMS tuning fork gyroscope is subjected to a Z-axis shock impact, the primary cause of failure is bending deformation of the connecting beam. The indirect non-contact displacement constraint improves the shock resistance by increasing the stiffness and the natural frequency of the gyroscope under the shock impact. This can increase the maximum allowable distance between the MEMS movable mass and the stop, avoiding the problem of adhesion and manufacturing techniques. When the MEMS tuning fork gyroscope is subjected to an X-axis shock impact, the collision of the oscillating frame of the MEMS tuning fork gyroscope with the displacement constraint may cause adverse consequences beyond prediction. Unlike the general one-sided collision, insufficient spacing between the displacement constraints and high-g shock impact will cause the small-stiffness oscillating frame to collide back and forth. The displacement of acceleration before the back-and-forth collision occurs is much larger than the displacement of the acceleration before the one-sided collision. Also, the initial velocity of the rebound after the initial collision will contribute to the velocity of the secondary collision. Persistent acceleration with an initial velocity and extended acceleration time will result in stresses caused by collisions exceeding the predictions based on general conditions. The acceleration of the oscillating frame couples the external shock impact, and the inappropriate spacing between the displacement constraints causes the collision between the frame and the stops to change from a one-side collision to a back-and-forth collision. The back-and-forth collision amplifies the stress generated by the collision, which make MEMS tuning fork gyroscopes more prone to failure.

## Figures and Tables

**Table 1:** Material properties used in the simulation.

Material	Young’s Modulus (GPa)	Poisson’s Ratio
Single-Crystal Silicon	130	0.28
Borosilicate Glass	64	0.2
